# MonitoraSB: an innovation for monitoring and strengthening oral health in primary health care in Brazil

**DOI:** 10.1590/1980-549720240065

**Published:** 2024-12-16

**Authors:** Raquel Conceição Ferreira, Loliza Chalub Luiz Figueiredo Houri, João Henrique Lara do Amaral, Maria Edileusa Santos, Elisa Lopes Pinheiro, Priscila Morais Gomes, Renata Maria Mendes de Oliveira, Rosana Leal do Prado, Najara Barbosa da Rocha, Hernane Braga Pereira, Jacqueline Silva Santos, Doralice Severo da Cruz, Maria Inês Barreiros Senna

**Affiliations:** I Universidade Federal de Minas Gerais, School of Dentistry, Department of Social and Preventive Dentistry – Belo Horizonte (MG), Brazil.; III State Health Department of Minas Gerais, Oral Health Department – Belo Horizonte (MG), Brazil.; IV Ministry of Health, Secretariat of Primary Health Care – Brasília (DF), Brazil.; V Universidade Federal de Minas Gerais, School of Dentistry, Department of Dental Clinic, Pathology and Surgery – Belo Horizonte (MG), Brazil.

**Keywords:** Health evaluation, Public health services, Health information systems, Health strategies

## Abstract

**Objective:**

This study describes the methodology used in the development of MonitoraSB, an innovation in the field of evaluating and monitoring oral health services in primary health care (PHC) in Brazil, and it discusses its characteristics and possible uses.

**Methods:**

MonitoraSB includes a matrix of indicators and digital tools, developed in collaboration with oral health team dentists and oral health managers. The indicators evaluate the provision and management of oral health services and were developed on the basis of the model for evaluating the effectiveness of oral health care. The dashboard and calculator were developed to facilitate the operational use of the indicators.

**Results:**

The 54 indicators in the matrix, with demonstrated content validity and measurability, cover various aspects of oral health care in PHC, from the organization and capacity of services to the profile of care and resolution. The dashboard offers navigation and interactivity, allowing regional, state and municipal analysis and making geographical and temporal comparisons. The calculator allows indicators to be obtained at the local level.

**Conclusion:**

The implementation of MonitoraSB could promote the strengthening of PHC, supporting informed decision-making and the consolidation of the universal, comprehensive and equitable oral health care model.

## INTRODUCTION

Primary health care (PHC), the gateway and structural basis of Unified Health System (SUS), is essential in managing health care^1^. Strengthening PHC is essential for the resilience of health systems^2^, ensuring equitable and quality care for the needs of the population. One of the strategies for this is to institutionalize monitoring and evaluation practices^3^. Monitoring corresponds to the systematic monitoring of services based on information about the reality^4^.

In Brazil, the institutionalization of PHC assessment processes began with the creation of the Coordination of Monitoring and Evaluation of the Primary Care Department in 2000^5^. Since then, several initiatives have been proposed by the Ministry of Health: Proposal for Evaluation of the Performance of the Health System (PROADESS)^6^, Evaluation for Improvement of the Quality of the Family Health Strategy^7^, Evaluation of the Quality of Primary Care Services^8^, Use of the PHC Evaluation Instrument (PCATool)^9^, Program for Improvement of Access and Quality of Primary Care (PMAQ-AB)^10^, development of the Unified Health System Performance Index (IDSUS)^11^ and reformulation of the National Program for Evaluation of Health Services (PNASS)^12^.

PHC is also the gateway to the National Oral Health Policy (PNSB), which guides the evaluation and monitoring of the results achieved, aiming to guide informed decision-making through indicators^13–17^. Oral health indicators were established in the Primary Care Indicators Pact (1998–2006), in the Health Pacts (2007–2011) and in the resolutions of the Tripartite Intermanagerial Commission (2012, 2013 and 2016)^18^. The IDSUS included indicators related to access to primary care. In the 3rd Cycle of the PMAQ-AB, oral health monitoring indicators assessed access, continuity of care, resolution and scope of services^10^. The Previne Brasil Program defined the indicator dental care for pregnant women to finance oral health teams (eSBs)^19^.

On April 10, 2024, a new methodology for federal co-financing of the PHC floor was established by the Ministry of Health^20^. One of the components of co-financing for eSBs aims to stimulate the achievement of agreed indicators, encouraging the improvement of access and quality of services in PHC^20^.

Despite these monitoring and evaluation initiatives, oral health indicators have only covered some dimensions of service quality and have suffered from discontinuity, which may hinder the provision of services to meet the population's needs^18,21^, constituting a problem that needs to be overcome^14^. Therefore, ensuring the availability, analysis and use of information on the population's health conditions and the health system's response capacity to qualify management and care is a priority, promoting equity and universal access to oral health^22^. Additionally, the use of digital information and data management technologies can enhance the use of this information in the formulation of health policies, contributing to the digital transformation of SUS^23^.

However, the fragmentation of monitoring systems and the lack of indicators to guide policies are recognized global challenges^24^. Recently, the World Health Assembly recommended the implementation of effective surveillance and monitoring systems^25^. Accordingly, the World Health Organization (WHO) launched the Global Oral Health Status Report on a digital portal^26^, providing a comprehensive overview of the global burden of oral diseases based on the most recent data from the Global Burden of Disease (GBD) project, the International Agency for Research on Cancer (IARC), and WHO research. Other digital dashboards, such as that of the Centers for Disease Control and Prevention^27^, were identified, and they present research results with available primary data. No international monitoring initiatives based on routine data from national health information systems were found.

Several initiatives have been developed in Brazil, such as the primary care indicator dashboards^28^, which provide indicators on PHC coverage, service production and family health, among others. These dashboards aim to promote knowledge about PHC, support decision-making and increase the transparency of the Department of Primary Health Care (SAPS), facilitating monitoring and evaluation. The LocalizaSUS panels, created by the Department of Monitoring, Evaluation and Dissemination of Strategic Health Information, of the Secretariat of Information and Digital Health (SEIDIGI) of the Ministry of Health, disseminate strategic data and information to managers, researchers and citizens. Oral health is already being developed in the PHC dashboards, but is not yet available in LocalizaSUS.

In this context, MonitoraSB was developed, a name for a proposal for monitoring oral health services in PHC, consisting of an evaluation matrix of indicators^29^ and new digital tools^30,31^. The development of this innovation aimed to contribute to changing the focus of evaluation in PHC, from achieving goals to the regular and continuous use of indicators for monitoring health services and planning at various levels of management, in addition to contributing to the process of monitoring oral health services in PHC in Brazil. This paper describes the methodology for developing MonitoraSB and discusses its characteristics and possible uses.

### Process of developing and detailing MonitoraSB

MonitoraSB includes a matrix of indicators and digital tools developed collaboratively by dentists working in eSB in PHC and oral health managers in Minas Gerais, at the municipal and state levels.

Matrix of indicators: the indicators were developed based on the model for assessing the effectiveness of oral health care adapted from Nickel^32^ and modified by Colussi^33^, in addition to the principles of the PNSB and the National Primary Care Policy. Two dimensions of quality of oral health services were considered, with subdimensions: oral health management (intersectoral action/popular participation, eSB work process) and provision of oral health services (access to oral health services, surveillance without oral health, promotion and prevention and diagnosis, treatment and rehabilitation in oral health)^32,33^. The indicators presented content validity and measurability with data from the Health Information System for Primary Care (SISAB)^29^. Details on the development of the indicators, including concepts of dimensions/subdimensions, have been presented in previous publications^29,34^.

The indicators are calculated using routine data made available by SISAB, which are generated through the registration of user care in the PHC. These data are collected by the two e-SUS PHC software systems: the Electronic Citizen Record or Simplified Data Collection (non-computerized municipality)^35^. In SISAB, the databases for the numerator and denominator of the indicators are obtained by consulting the health reports: production and collective activity. Data on the registered population are obtained from the registration panel in this same system. An open-access indicator dictionary presents each indicator's qualification sheets regarding measurement, interpretation, uses, limitations, calculation method, data source and parameter^34^.

Digital tools: MonitoraSB includes two complementary digital tools to operationalize the use of indicators by services: a dashboard and an indicator calculator. The dashboard presents indicators for Brazil, macro-regions, 27 federative units (FUs) and municipalities, according to the data available in SISAB. The construction involved a data scientist with a degree in Systems Engineering, and followed six steps:

1. Creation of a spreadsheet describing the calculation method, treatment of missing data and filters of the reports to be selected to obtain the numerator and denominator of the indicators;
2. Identification of the numerator and denominator databases to be extracted for the calculation;
3. Double validation of the extraction and calculation methods by the research team and the data scientist. In this stage, the researchers performed manual extraction, linked the databases, and calculated the indicators using Stata 18.0 software. The data scientist carried out the same process to identify inconsistencies and standardize the process;
4. Automation of file extraction and calculation of indicators, creating scripts using Python^®^ language;
5. Storage of the history of calculated indicators in an analytical database, that is, creation of the data infrastructure, a delta lake composed of the following data layers: Landing zone; Bronze layer; Silver layer; Gold layer;
6. Creation of the dashboard using data contained in the gold layer and making the calculated indicators available on the interactive dashboard for users.

The team defined the characteristics of the dashboard in terms of structure and organization (header, colors, access to the indicator qualification sheet — interactive exploration of the dashboard — drill-through), navigation and interactivity (navigation sidebar by dimension and, separately, for each indicator; presentation of indicators by region, FU and municipality; inclusion of annual and quarterly temporal filters (minimum period of data availability in SISAB at the time of development); presentation of results in graphs or maps for geographic and temporal comparisons; inclusion of socioeconomic (municipal Human Development Index; HDI) and demographic (population size) filters, enabling analysis of disparities in the results. For these last two filters, the population estimate databases made available by IBGE in each year and the HDI database, obtained by accessing the Human Development Atlas, were used. A pre-test of the dashboard's usability was carried out with five PHC dentists, resulting in changes to the design, specifically in the presentation of indicators by region and state, which were separated from the detailed information by municipality.

The calculator allows you to obtain indicators for each eSB using data from e-SUS PHC reports generated locally by managers or professionals. It was developed in Python^®^ using the open-source tool Streamlit, which allows you to create and host simple applications on the internet for free.

To illustrate the functionality of the dashboard and calculator, the results were shown for the indicator "Coverage of first programmatic dental consultation", from the subdimension "Access to oral health services", which measures the number of first programmatic dental consultations in the PHC in a given location and period, per registered population, in the same location and period (per thousand users).

### Evaluation matrix

The evaluation matrix is composed of 54 indicators measurable through SISAB data and 15 validated and non-measurable with the available data, in the format current by the system, in April 2024. The objectives of the indicators were described in Chart 1 and the indicators measurable with SISAB data in Chart 2.

**Chart 1:** Number of indicators and measurement objectives of the set and indicators of a given sub-dimension/dimension of monitoring oral health services in primary health care.

Dimensions/subdimensions		Objectives for measuring the indicators of each subdimension
Provision of oral health services		
	Access to oral health services (9 indicators)	Contribute to monitoring and evaluating the organization and capacity of oral health services to provide objective responses to the problems presented by users. They also contribute to estimating the coverage of actions, the profile of care and the resolution of individual oral health care.
	Oral health monitoring (5 indicators)	These can reveal the health-disease process at the population level through the frequency of morbidity treated by eSBs for selected conditions (toothache, dentoalveolar abscess, soft tissue changes, and cleft lip and palate). They can indicate the oral health conditions of the registered population, in addition to supporting planning, management, and evaluation processes of health services/actions and providing opportunities for the implementation of public policies to protect the health of the population.
	Oral health diagnosis, treatment and rehabilitation (16 indicators)	Estimate the frequency of certain types of individual dental procedures and referrals to other eSF professionals for specialized oral health care and diagnostic support services. These estimates can indirectly indicate the profile of oral health needs of the population, as well as the work process of the team, contributing to the monitoring and evaluation of the oral health care model and the level of organization of the care network.
	Promotion and prevention (14 indicators)	They estimate the provision of individual preventive procedures and the implementation and coverage of collective oral health activities (educational, group care, collective practices). These indicators contribute to the evaluation of the work process of the eSB and the oral health care model developed in the area.
Oral health management		
	Intersectoral action/popular participation (4 indicators)	They estimate the performance of Primary Health Care Teams in collective activities and the participation of the community in these activities. These indicators contribute to assessing how much the work process of the eSFs encourages popular participation and seeks to establish intersectoral collaboration in their territory of operation.
	eSB work process (6 indicators)	They assess the performance and protagonism of eSBs in the daily multidisciplinary work in PHC. These indicators also contribute to highlighting the themes/activities related to collaborative work in the ESF under the leadership of eSB professionals.

eSB: oral health team; ESF: Family Health Team; PHC: primary health care.

**Chart 2:** Indicators for monitoring oral health services in Primary Health Care by theoretical dimension/subdimension.

Indicators*	
Provision of oral health services	
	Access to oral health services
	Proportion of scheduled appointments performed at the UBS
	Proportion of pregnant women with dental care provided
	Ratio between spontaneous demand appointments and scheduled appointments
	Proportion of emergency dental care in spontaneous demand
	Rate of emergency dental care per registered population
	Average number of return dental appointments per completed treatment
	Coverage of first programmatic dental appointment
	Ratio between completed treatment and first programmatic dental appointments
	Rate of dental surgeon appointments per registered population
Oral health surveillance	
	Dental care rate for toothache
	Proportion of users with toothache treated in the emergency room
	Dental care rate for dentoalveolar abscess
	Dental care rate for soft tissue changes
	Dental care rate for users with cleft lip and palate
Diagnosis, treatment and rehabilitation in oral health	
	Proportion of emergency dental care
	Proportion of clinical-surgical dental procedures
	Proportion of restorative dental procedures
	Proportion of permanent tooth extractions in dental procedures
	Average number of adaptation procedures per installed prosthesis
	Ratio between appointments for other PHC professionals and care provided by the eSB
	Average number of referrals to OMF surgery
	Average number of referrals to endodontics
	Average number of referrals to oral medicine
	Average number of referrals to implantology
	Average number of referrals to pediatric dentistry
	Average number of referrals to orthodontics/orthopedics
	Average number of referrals to periodontics
	Average number of referrals to dental prosthetics
	Average number of referrals to radiology
	Diagnostic support in radiology for dental procedures
Promotion and prevention	
	Proportion of individual preventive procedures in oral health
	Scheduling of users for group activities by the Oral Health Team
	Group care activities
	Health education activities
	Collective assessment/procedure activities
	Educational activities for children in early childhood (0 to 3 years)
	Educational activities for preschool children (4 and 5 years)
	Educational actions for school-age children (6 to 11 years)
	Educational action for tobacco control
	Rate of user participation in health education activities
	Rate of user participation in group care
	Rate of user participation in collective assessment/procedure activities
	Collective practices in oral health
Oral health topics in collective activities	
	Oral health management
	Intersectoral action/popular participation
	Proportion of meetings for participatory planning and evaluation
	Proportion of social mobilization activities
	Degree of social participation in relation to health education activities
	Proportion of collective activities directed at education professionals
eSB work process	
	Degree of protagonism of eSB in team meetings
	Degree of organization of eSB in relation to the team's work process
	Degree of organization of eSB in relation to administrative/operational issues
	Degree of organization of eSB in relation to the diagnosis and monitoring of the area
	Degree of organization of eSB in relation to the discussion of cases and individual therapeutic projects
	Degree of organization of eSB in relation to continuing education

* The qualification sheets for each indicator can be found in the Dictionary of Indicators (https://pergamum.bu.ufmg.br/pergamumweb/vinculos/00002d/00002d44.pdf).

UBS: basic health unit; PHC: primary health care; eSB: oral health team; OMF: oral and maxillofacial.

### Indicator dashboard

The indicator panel, registered with the National Institute of Industrial Property (BR 51 2023 002193-8), is available for implementation at the link https://lookerstudio.google.com/reporting/86c09403-f4a0-4625-ad1f-239daa77f6a2/page/p_rpxxv2ub8c ^30^. It is worth noting that there are possibilities of integrating MonitoraSB with other monitoring systems in force in Brazil. The panel is in the process of being incorporated by the General Coordination of Oral Health of the Ministry of Health (CGSB) as a tool to be recommended for monitoring oral health services in Brazil and will be hosted in the Strategic Management Room (LocalizaSUS/SEIDIGI). Indicators from the evaluation matrix are also expected to make up the oral health panel, which will integrate with the PHC monitoring system in the panels under construction.

**Figure 1:**
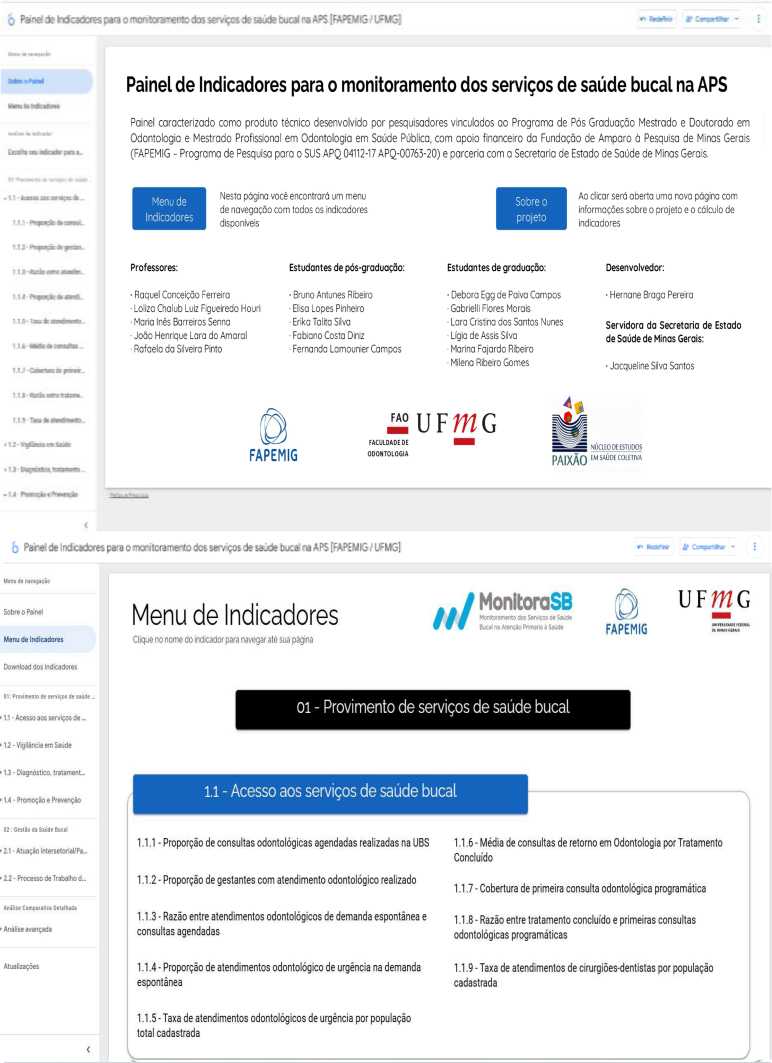
Navigation structure of the indicator dashboard for monitoring oral health services in primary health care. (*Figure was not translated and left in original Portuguese.*)

**Figure 2:**
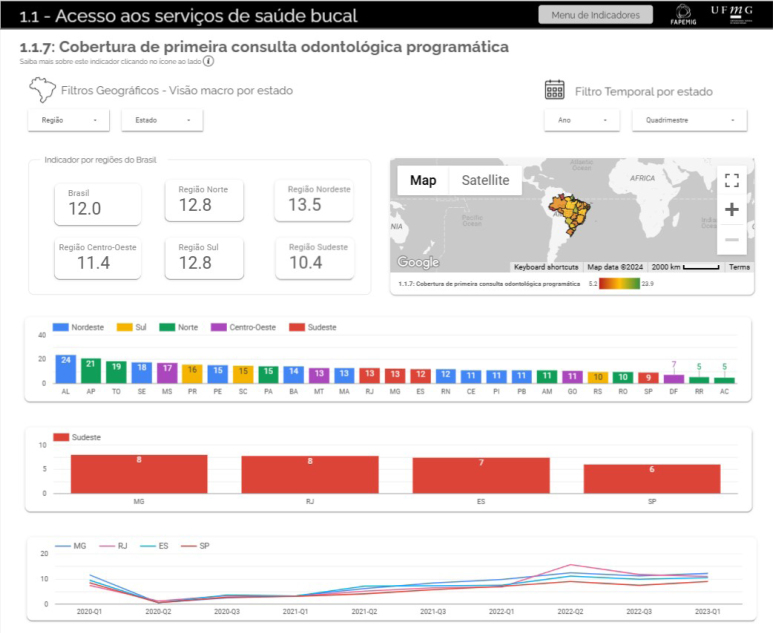
Viewing the results of the indicator "Coverage of first programmatic dental consultation" with geographic filters by region and Brazilian federative units and temporal filters. (*Figure was not translated and left in original Portuguese.*)

The dashboard has navigation bars on the home page and on the sidebar, allowing quick and interactive navigation from one indicator to another (Figure 1). Navigation by the selected indicator can be performed, obtaining a view by region or state, according to the desired time filter. For example, Figure 2 illustrates the results of the indicator "Coverage of first programmatic dental consultation" for the year 2023, quarter 1 (Figure 2a).

The visualization shows the results for Brazil (12 dental appointments per thousand users) and for each region, with values ranging from 13.5 in the Northeast to 10.4 in the Southeast (per thousand users). The Brazil map shows each state's indicator values, representing a color scale from the lowest to the highest value. The results can also be viewed for each state in bar graphs, allowing comparison and analysis of geographic disparities in the indicators. For example, for this indicator, the lowest results were observed in Roraima and Acre, and the highest in Alagoas (Figure 2a). A detailed view can be seen by selecting a specific region (Figure 2b). When more than one four-month period is selected, the panel also allows temporal comparisons, that is, variations in the indicator over time, according to the selected geographic area. For example, Figure 2b shows the variation in the indicator since the first four-month period of 2020 for each of the states in the Southeast Region (Figure 2b). In this case, lower values of the indicator are observed in the second four months of 2020, coinciding with the interruption of dental services due to the COVID-19 pandemic. Temporal analysis can be useful for assessing trends, effects of health actions, policies and programs, and for resource planning. It is essential for monitoring services and understanding their functioning and response during events such as the pandemic or other situations that require resilience and the ability to adapt and reorganize services.

**Figure 3:**
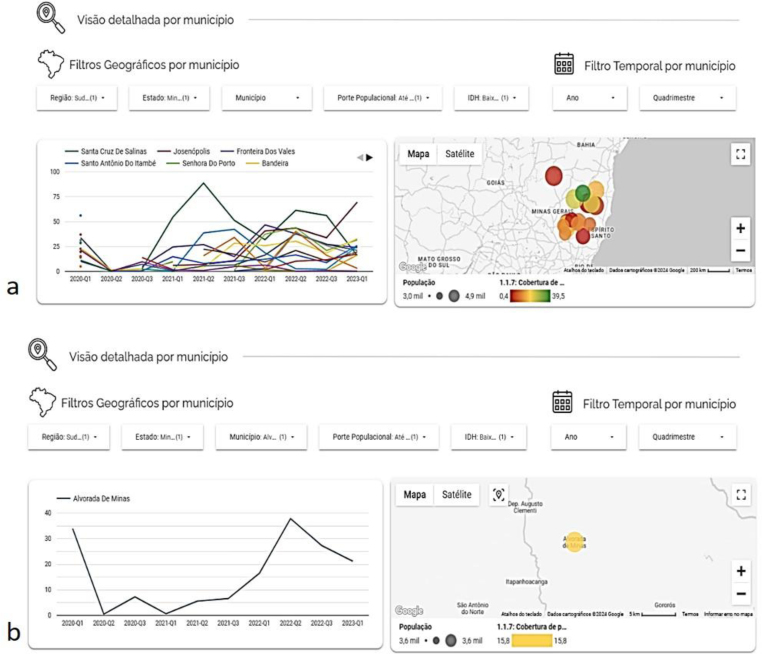
Visualization of the results of the temporal variation of the indicator "Coverage of first programmatic dental consultation" at the municipal level, considering filters of population size and municipal Human Development Index. (*Figure was not translated and left in original Portuguese.*)

The analysis by municipality makes it possible to compare indicators according to geographic, demographic or socioeconomic characteristics (Figure 3). For example, Figure 3 illustrates the results of the indicator "Coverage of first programmatic dental consultation" for municipalities in Minas Gerais with up to 5,000 inhabitants and a very low HDI. In this example, the periods between the four-month periods of 2020Q1 and 2023Q1 were selected for temporal evaluation, evidencing a similar pattern of reduction in the indicator in 2020Q2 and many fluctuations, but with a pattern of increase in the number of first programmatic dental consultations for every thousand registered users in many municipalities after 2020Q2.

It was also observed that the indicator was not calculated in some municipalities in 2020Q1 and 2020Q2, evidenced by the timeline's interruption. In these municipalities and four-month periods, there was no record of first programmatic dental consultation. The heat map shows the municipalities with the greatest coverage in colors close to green and those with the lowest indicator values in red. It is worth noting that the map shows the indicator calculated considering the selected period, in the example, for all four-month periods. In addition, the diameter of the circle represents the population size of the municipality. The HDI filter could be used for comparative analysis of municipalities with other HDI values (Figure 3a).

An analysis can be performed by selecting only the municipality. In the example, Alvorada de Minas presented lower indicator values in the periods between 2020Q1 and 2021Q1, with a positive variation since this four-month period (Figure 3b). The dashboard is also a report and database generator, as it allows the download of calculated indicators in spreadsheets for the disaggregation levels of Brazil, region, state and municipality.

### Calculator of indicators

**Figure 4:**
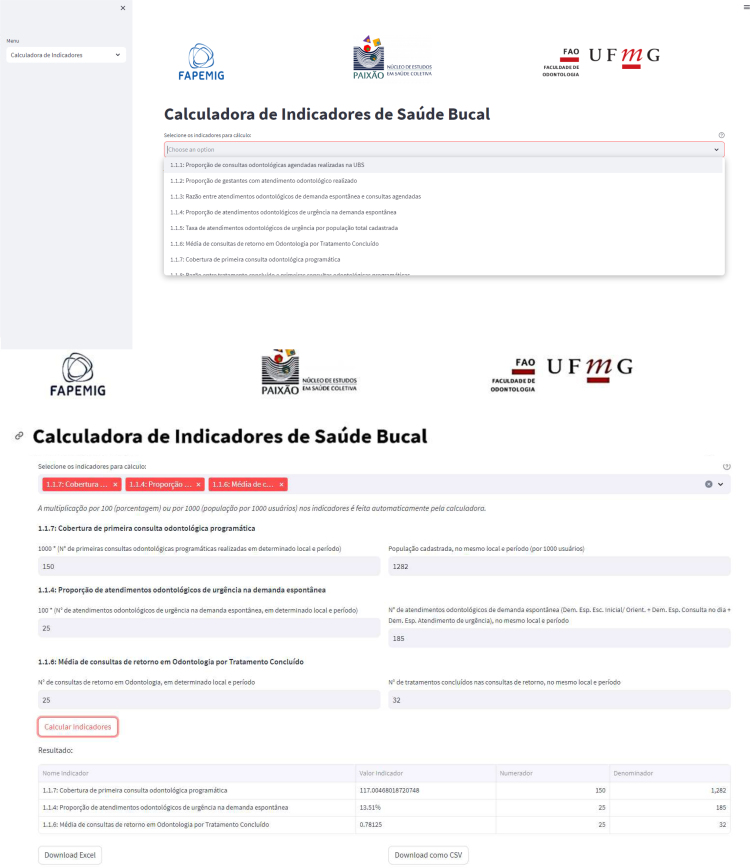
Visualization of the steps for determining oral health indicators using the calculator. (*Figure was not translated and left in original Portuguese.*)

The oral health indicator calculator is an open-access online tool (bit.ly/calculadora-indicadores-saude-bucal-ppsus). When accessing the calculator, the user will select the indicator they wish to calculate. Then boxes will appear on the screen to enter each indicator's numerator and denominator data. This data should be obtained from the e-SUS PHC management reports for each eSB. After entering the data and clicking on "Calculate Indicators", the results will be displayed. The user will also have the option to download the results in Excel or *csv format (Figure 4).

### MonitoraSB: implications for monitoring and evaluating oral health services

This paper presents an innovative proposal for monitoring and evaluating public oral health services by developing a matrix of indicators and proposing two new, freely accessible digital tools to operationalize it. The matrix, based on national health production data, guarantees the availability of information^36^, guiding political decisions and subsidizing social control. The indicators address various aspects of oral health care, from the coverage of services in PHC, both individual and collective, and allow for an understanding of the demand for specialized services, the predominant care model and the rates of care for specific conditions^29^.

By expanding the scope of indicators related to the provision and management of oral health, the matrix offers new possibilities compared to existing initiatives until now, which focused on data related to access to and coverage of oral health services and which have been reduced over the years^18,21^.

The digital tools operationalize the calculation of indicators and their use by services. By allowing temporal and spatial cuts, the panel facilitates the comparison of results at multiple levels and favors the continuous monitoring of oral health services, allowing for the evaluation, transparency and continuous reorientation of interventions.

As it uses the SUS information systems, it offers a perspective based on reality and enables the qualification of records. This tool also strengthens the use of health information systems, aligning with the implementation of SUS Digital Brasil^23^. By including data fed by all Brazilian municipalities and being an open tool, the MonitoraSB panel democratizes access to information.

As they use Delta Lake in their architecture for data extraction and storage, digital tools favor an innovative ecosystem for storing large volumes and multiple forms of information processing^37,38^. This favors the timeliness of information accessed by users through the provision of new data by SISAB and ensures that the right information is available at the right time and in the right place for evidence-based decision-making^39^.

With a user-friendly interface, the dashboard allows quick visualization of results and multiple comparisons over time by region, population size, and municipal human development index bands. Thus, after selecting the indicator, the tool offers a certain degree of customization and automatic visual resources. Considering the health situation room as a space for systematic analysis of information^40^, this tool can be understood as such or even as support for local technical teams, providing continuous monitoring of indicators for planning and rapid identification of non-standard occurrences^41^, assisting in oral health surveillance. In addition to the existing customization, it is possible to calculate indicators individually with the digital calculator, allowing for more specific cuts. These tools help to overcome the fragmentation of oral health data and seek equity in access to information and technologies. These aspects are essential to improve PHC and increase its resilience, especially in a country with great inequalities in the use and access to technologies by professionals and where digital exclusion is still prominent^2^.

However, some challenges must be faced, including the need to resize the indicator matrix, which the use of tools in real contexts should facilitate. On the other hand, the expanded scope of indicators can contribute to changing the monitoring and evaluation models that previously existed in Brazil, which are often linked to meeting targets linked to financial transfers.

From this perspective, MonitoraSB offers possibilities for integration with existing monitoring systems, especially through CGSB and SEIDIGI, as already mentioned. In addition, some of its features, such as the use of data science in its development and the possibility of obtaining information by the municipality in *csv format, also favor interoperability with initiatives such as PROADESS. In this case, one of the possibilities is using health system performance indicators containing geospatial information to understand oral health indicators better^42^.

Another challenge is the source of the data, which affects the quality of the records in the information systems, which may contain inconsistencies, conflicting information, missing data or even being affected by under-recording. Given its nature, the lowest level of disaggregation of the indicators in the panel is at the municipal level. However, the vast majority of Brazilian municipalities are small, around 70% of which have less than 20 thousand inhabitants, and tools like this can offer significant progress in their management^43^. The calculator sought to overcome this limitation.

Adopting digital tools in PHC health services is a challenge to overcome. An implementation study in municipal contexts is underway to assess the acceptability, adoption, feasibility, suitability, and sustainability of SUS professionals’ use of MonitoraSB. This study also analyzed its impact on the outcomes of health services, observing the variation of the indicators themselves over time and the quality of data recording in e-SUS PHC. It is also expected that the use of indicators in different contexts will allow for the discussion and definition of acceptable or desirable quality parameters for their measurement.

MonitoraSB provides indicators to monitor the oral health of SUS users, improving health planning and contributing to the advancement of public policies. It provides dynamic and real-time information for managers, academics, social movements and decision-makers. It is expected that the implementation of MonitoraSB will promote the strengthening of PHC and PNSB, contributing to the consolidation of a universal, comprehensive and equitable oral health care model.
