# Supplementary material for: MonitoraSB: an innovation for monitoring and strengthening oral health in primary health care in Brazil
Source: Rev Bras Epidemiol. 2024 Dec 16;27:e240065. doi: 10.1590/1980-549720240065 (PMC11654291; doi:10.1590/1980-549720240065)
Supplement: Translation

# Translation: MonitoraSB: uma inovação para o monitoramento e o fortalecimento da saúde bucal na Atenção Primária à Saúde no Brasil

**DOI:** 10.1590/1980-549720240065.2

**Authors:** Raquel Conceição Ferreira, Loliza Chalub Luiz Figueiredo Houri, João Henrique Lara do Amaral, Maria Edileusa Santos, Elisa Lopes Pinheiro, Priscila Morais Gomes, Renata Maria Mendes de Oliveira, Rosana Leal do Prado, Najara Barbosa da Rocha, Hernane Braga Pereira, Jacqueline Silva Santos, Doralice Severo da Cruz, Maria Inês Barreiros Senna

**Affiliations:** I Universidade Federal de Minas Gerais, Faculdade de Odontologia, Departamento de Odontologia Social e Preventiva – Belo Horizonte (MG), Brasil.; III Secretaria de Estado de Saúde de Minas Gerais, Diretoria de Saúde Bucal – Belo Horizonte (MG), Brasil.; IV Ministério da Saúde, Secretaria de Atenção Primária à Saúde – Brasília (DF), Brasil.; V Universidade Federal de Minas Gerais, Faculdade de Odontologia, Departamento de Clínica, Patologia e Cirurgia Odontológicas – Belo Horizonte (MG), Brasil.

**Keywords:** Avaliação em saúde, Serviços públicos de saúde, Sistemas de informação em saúde, Estratégias de saúde

## Abstract

**Objetivo:**

Este estudo descreveu a metodologia utilizada no desenvolvimento do MonitoraSB, uma inovação no campo da avaliação e do monitoramento dos serviços de saúde bucal na Atenção Primária à Saúde (APS) no Brasil e discutiu suas características e possíveis utilizações.

**Métodos:**

O MonitoraSB inclui uma matriz de indicadores e ferramentas digitais desenvolvidas em colaboração com cirurgiões-dentistas das equipes de saúde bucal e gestores de saúde bucal. Os indicadores avaliam o provimento e a gestão dos serviços de saúde bucal e foram desenvolvidos com base no modelo de avaliação da efetividade da atenção em saúde bucal. O painel e a calculadora foram desenvolvidos para facilitar o uso operacional dos indicadores.

**Resultados:**

Os 54 indicadores da matriz, com validade de conteúdo e mensurabilidade demonstradas, abrangem diversos aspectos do cuidado em saúde bucal na APS, desde a organização e a capacidade dos serviços até o perfil de atendimento e resolutividade. O painel oferece navegação e interatividade, permitindo análises nos níveis regional, estadual e municipal, bem como comparações geográficas e temporais. A calculadora permite a obtenção dos indicadores no nível local.

**Conclusão:**

A implementação do MonitoraSB poderá fortalecer a APS, sustentando a tomada de decisões informadas, e consolidar o modelo de cuidado em saúde bucal universal, integral e equânime.

## INTRODUÇÃO

A Atenção Primária à Saúde (APS), porta de entrada e base estruturante do Sistema Único de Saúde (SUS), é fundamental na gestão do cuidado à saúde^1^. Fortalecer a APS é primordial para a resiliência dos sistemas de saúde^2^, garantindo atendimento equitativo e de qualidade às necessidades da população. Uma das estratégias para isso é institucionalizar práticas de monitoramento e avaliação^3^. O monitoramento corresponde ao acompanhamento sistemático dos serviços com base em informações sobre a realidade^4^.

No Brasil, a institucionalização dos processos avaliativos da APS iniciou-se com a criação da Coordenação de Acompanhamento e Avaliação do Departamento de Atenção Básica, em 2000^5^. Desde então, várias iniciativas foram propostas pelo Ministério da Saúde: Proposta de Avaliação do Desempenho do Sistema de Saúde (PROADESS)^6^, Avaliação para Melhoria da Qualidade da Estratégia Saúde da Família^7^, Avaliação da Qualidade de Serviços de Atenção Básica^8^, Uso do Instrumento de Avaliação da APS (PCATool)^9^, Programa para Melhoria do Acesso e da Qualidade da Atenção Básica (PMAQ-AB)^10^, desenvolvimento do Índice de Desempenho do Sistema Único de Saúde (IDSUS)^11^ e reformulação do Programa Nacional de Avaliação dos Serviços de Saúde (PNASS)^12^.

A APS é também a porta de entrada na Política Nacional de Saúde Bucal (PNSB), que orienta a avaliação e o monitoramento dos resultados alcançados, visando orientar tomadas de decisão informadas por meio de indicadores^13–17^. Indicadores de saúde bucal foram estabelecidos no Pacto de Indicadores da Atenção Básica (1998–2006), nos Pactos pela Saúde (2007–2011) e nas resoluções da Comissão Intergestores Tripartite (2012, 2013 e 2016)^18^. O IDSUS incluiu indicadores referentes ao acesso à atenção básica. No 3° Ciclo do PMAQ-AB, indicadores de monitoramento da saúde bucal avaliaram acesso, continuidade do cuidado, resolutividade e abrangência dos serviços^10^. O Programa Previne Brasil definiu o indicador atendimento odontológico a gestantes para financiamento das equipes de saúde bucal (eSBs)^19^.

Em 10 de abril de 2024, foi instituída nova metodologia de cofinanciamento federal do piso de APS, pelo Ministério da Saúde^20^. Um dos componentes do cofinanciamento para as eSBs visa estimular o alcance dos indicadores pactuados, incentivando a melhoria do acesso e da qualidade dos serviços na APS^20^.

Apesar dessas iniciativas de monitoramento e avaliação, os indicadores de saúde bucal contemplaram apenas algumas dimensões da qualidade dos serviços e sofreram descontinuidade, o que pode prejudicar o atendimento às necessidades da população^18,21^, constituindo um problema a ser superado^14^. Portanto, é prioritário garantir disponibilidade, análise e uso da informação sobre as condições de saúde da população e a capacidade de resposta do sistema de saúde para qualificar a gestão e a atenção, promovendo a equidade e o acesso universal à saúde bucal^22^. Adicionalmente, o uso de tecnologias digitais de informação e gestão de dados pode potencializar o emprego dessas informações na formulação de políticas de saúde, contribuindo para a transformação digital no SUS^23^.

Contudo, a fragmentação dos sistemas de monitoramento e a falta de indicadores para orientar políticas são desafios globais reconhecidos^24^. Recentemente, a Assembleia Mundial da Saúde recomendou a implementação de sistemas de vigilância e monitoramento eficazes^25^. Nesse contexto, a Organização Mundial da Saúde (OMS) lançou o Relatório Global de Estado de Saúde Bucal em um portal digital^26^, fornecendo uma visão abrangente da carga global das doenças bucais com base nos dados mais recentes do projeto *Global Burden of Disease* (GBD), da Agência Internacional de Pesquisa sobre o Câncer (IARC) e de pesquisas da OMS. Foram identificados outros painéis digitais, como o do Centro para Prevenção e Controle de Doenças^27^, que apresentam resultados de pesquisas com dados primários disponíveis. Não foram encontradas iniciativas internacionais de monitoramento baseado em dados rotineiros de sistemas nacionais de informação de saúde.

Diversas iniciativas têm sido desenvolvidas no Brasil, tais como os painéis de indicadores da atenção básica^28^, que disponibilizam indicadores sobre cobertura da APS, e a produção de serviços e saúde da família, dentre outras. Esses painéis visam promover o conhecimento sobre a APS, subsidiar a tomada de decisão e aumentar a transparência da Secretaria de Atenção Primária à Saúde (SAPS), facilitando o monitoramento e a avaliação. Os painéis LocalizaSUS, criados pelo Departamento de Monitoramento, Avaliação e Disseminação de Informações Estratégicas em Saúde, da Secretaria de Informação e Saúde Digital (SEIDIGI) do Ministério da Saúde, disseminam dados e informações estratégicas para gestores, pesquisadores e cidadãos. A saúde bucal já está em desenvolvimento nos painéis da APS, mas ainda não está disponível no LocalizaSUS.

Nesse contexto, foi proposto o MonitoraSB, denominação para uma proposta de monitoramento dos serviços de saúde bucal na APS composta por uma matriz avaliativa de indicadores^29^ e ferramentas digitais inéditas^30,31^. O desenvolvimento dessa inovação visou contribuir para a mudança do enfoque da avaliação na APS, do alcance de metas para o uso regular e contínuo de indicadores para acompanhamento dos serviços de saúde e planejamento nos vários níveis de gestão, além de contribuir para o processo de vigilância dos serviços de saúde bucal na APS no Brasil. Esse trabalho descreve a metodologia de desenvolvimento do MonitoraSB e discute suas características e possíveis utilizações.

### O percurso de elaboração e detalhamento do MonitoraSB

O MonitoraSB inclui uma matriz de indicadores e ferramentas digitais elaborados, colaborativamente, por cirurgiões-dentistas atuantes em eSB na APS e gestores de saúde bucal em Minas Gerais, nos níveis municipal e estadual.

Matriz de indicadores*:* os indicadores foram desenvolvidos com base no modelo de avaliação da efetividade da atenção em saúde bucal adaptado de Nickel^32^ e modificado por Colussi^33^, além dos princípios da PNSB e da Política Nacional de Atenção Básica. Duas dimensões de qualidade dos serviços de saúde bucal foram contempladas, com subdimensões: gestão de saúde bucal (atuação intersetorial/participação popular, processo de trabalho das eSBs) e provimento de serviços de saúde bucal (acesso aos serviços de saúde bucal, vigilância sem saúde bucal, promoção e prevenção e diagnóstico, tratamento e reabilitação em saúde bucal)^32,33^. Os indicadores apresentaram validade de conteúdo e mensurabilidade com dados do Sistema de Informação em Saúde para a Atenção Básica (SISAB)^29^. Detalhes sobre o desenvolvimento dos indicadores, incluindo conceitos das dimensões/subdimensões, foram apresentados em publicações prévias^29,34^.

Os indicadores são calculados usando dados rotineiros disponibilizados pelo SISAB, gerados por meio do registro dos atendimentos dos usuários na APS. A coleta desses dados é feita pelos dois sistemas de *softwares* do e-SUS APS: o Prontuário Eletrônico do Cidadão ou Coleta de Dados Simplificados (município não informatizado)^35^. No SISAB, as bases de dados do numerador e do denominador dos indicadores são obtidas por consulta aos relatórios de saúde: produção e atividade coletiva. Dados sobre a população cadastrada são obtidos do painel de cadastro nesse mesmo sistema. Um dicionário de indicadores, de acesso aberto, apresenta as fichas de qualificação de cada indicador quanto à medida, interpretação, usos, limitações, método de cálculo, fonte de dados e parâmetro^34^.

Ferramentas digitais: o MonitoraSB inclui duas ferramentas digitais complementares para operacionalizar o uso de indicadores pelos serviços: um painel e uma calculadora de indicadores. O painel apresenta indicadores para o Brasil, macrorregiões, 27 unidades da federação (UFs) e municípios, de acordo com a disponibilização dos dados no SISAB. A construção contou com um cientista de dados, com formação em Engenharia de Sistemas, seguiu seis etapas:

1. Criação de uma planilha descrevendo o método de cálculo, tratamento dos dados faltantes e dos filtros dos relatórios a serem selecionados para obtenção do numerador e do denominador dos indicadores;
2. Identificação das bases de dados do numerador e denominador a serem extraídas para o cálculo;
3. Dupla validação da forma de extração e do método de cálculo pela equipe de pesquisadores e pelo cientista de dados. Nessa etapa, os pesquisadores fizeram a extração manual, a vinculação das bases de dados e o cálculo dos indicadores usando o *software* Stata 18.0. O mesmo processo foi realizado pelo cientista de dados para identificar inconsistências e padronizar o processo;
4. Automatização da extração de arquivos e do cálculo de indicadores, criando *scripts* usando linguagem Python^®^;
5. Armazenamento do histórico de indicadores calculados em banco de dados analítico, ou seja, criação da infraestrutura de dados, um *delta lake* composto pelas camadas de dados: *Landing zone;* Camada bronze; Camada *silver;* Camada *gold*;
6. Criação do painel por meio dos dados contidos na camada *gold e* disponibilização dos indicadores calculados no painel interativo para os usuários.

A equipe definiu as características do painel quanto à estrutura e à organização (cabeçalho, cores, acesso à ficha de qualificação dos indicadores — exploração interativa do painel — *drill-through*), navegação e interatividade (barra lateral de navegação por dimensão e, separadamente, para cada indicador; apresentação dos indicadores por região, unidade da federação (UF) e município; inclusão de filtro temporal anual e quadrimestral (período mínimo de disponibilização dos dados no SISAB no momento do desenvolvimento); apresentação dos resultados em gráficos ou mapas para comparações geográficas e temporais; inclusão de filtros socioeconômicos (Índice de Desenvolvimento Humano Municipal) e demográficos (porte populacional), possibilitando analisar disparidades nos resultados. Para esses dois últimos filtros, foram empregadas as bases de dados de estimativas populacionais disponibilizadas pelo IBGE em cada ano e a base do Índice de Desenvolvimento Humano Municipal, obtida por acesso ao Atlas de Desenvolvimento Humano. Um pré-teste de usabilidade do painel foi realizado com cinco cirurgiões-dentistas da APS, resultando em mudanças no *design*, especificamente na apresentação dos indicadores por regiões e UF, que foram separados das informações detalhadas por municípios.

A calculadora permite obter indicadores para cada eSB utilizando dados dos relatórios e-SUS APS gerados localmente pelos gestores ou pelos profissionais. Foi desenvolvida em *Python* ^®^ e usando a ferramenta *open source Streamlit*, que permite criar e hospedar gratuitamente aplicações simples na internet.

Para ilustrar as funcionalidades do painel e da calculadora, foram demonstrados os resultados para o indicador "Cobertura de primeira consulta odontológica programática", da subdimensão "Acesso aos serviços de saúde bucal", que mensura o número de atendimentos de primeira consulta odontológica programática na APS em determinado local e período, por população cadastrada, no mesmo local e período (por mil usuários).

### Matriz avaliativa

A matriz avaliativa é composta por 54 indicadores mensuráveis por meio de dados do SISAB e 15 validados e não mensuráveis com os dados disponíveis, no formato vigente pelo sistema, em abril de 2024. Os objetivos dos indicadores foram descritos no Quadro 1 e os indicadores mensuráveis com dados do SISAB no Quadro 2.

**Quadro 1:** Número de indicadores e objetivos de mensuração do conjunto e indicadores de dada subdimensão/dimensão de monitoramento dos serviços de saúde bucal na Atenção Primária à Saúde.

Dimensões/subdimensões		Objetivos de mensuração dos indicadores de cada subdimensão
Provimento dos serviços de saúde bucal		
	Acesso aos serviços de saúde bucal (9 indicadores)	Contribuem para o monitoramento e a avaliação da organização e da capacidade dos serviços de saúde bucal em dar respostas objetivas aos problemas demandados pelos usuários. Contribuem também para estimar a cobertura das ações, o perfil do atendimento e a resolutividade da atenção individual em saúde bucal.
	Vigilância em saúde bucal (5 indicadores)	Podem revelar o processo saúde-doença em âmbito populacional por meio da frequência de morbidade atendida pelas eSBs de agravos selecionados (dor de dente, abscesso dentoalveolar, alteração em tecido mole e fendas ou fissuras labiopalatais). Podem indicar as condições de saúde bucal da população cadastrada, além de subsidiar processos de planejamento, gestão e avaliação dos serviços/ações de saúde e oportunizar a implementação de políticas públicas para a proteção da saúde da população.
	Diagnóstico, tratamento e reabilitação em saúde bucal (16 indicadores)	Estimam a frequência de determinados tipos de procedimentos odontológicos individuais e encaminhamentos para outros profissionais da eSF para atenção especializada em saúde bucal e serviços de apoio diagnóstico. Essas estimativas podem indicar indiretamente o perfil de necessidades em saúde bucal da população, bem como o processo de trabalho da equipe contribuindo para o monitoramento e a avaliação do modelo de atenção em saúde bucal e o nível de organização da rede de atenção.
	Promoção e prevenção (14 indicadores)	Estimam a oferta de procedimentos preventivos individuais e a execução e a cobertura de atividades coletivas em saúde bucal (educativas, atendimento em grupo, práticas coletivas). Esses indicadores contribuem para a avaliação do processo de trabalho das eSB e do modelo de atenção à saúde bucal desenvolvido no território.
Gestão da saúde bucal		
	Atuação intersetorial/participação popular (4 indicadores)	Estimam a atuação das Equipes de Atenção Primária à Saúde em atividades coletivas e a participação da comunidade nessas atividades. Esses indicadores contribuem para avaliar quanto o processo de trabalho das eSFs fomenta a participação popular e busca o estabelecimento de colaboração intersetorial no seu território de atuação.
	Processo de trabalho da eSB (6 indicadores)	Avaliam a atuação e o protagonismo das eSBs no cotidiano do trabalho multiprofissional na APS. Esses indicadores contribuem para apontar também os temas/atividades relacionados ao trabalho colaborativo na ESF sob liderança dos profissionais da eSB.

eSB: equipe de saúde bucal; ESF: Equipe Saúde da Família; APS: Atenção Primária à Saúde.

**Quadro 2:** Indicadores para o monitoramento dos serviços de saúde bucal na Atenção Primária à Saúde por dimensão/subdimensão teórica.

Indicadores*	
Provimento dos serviços de saúde bucal	
	Acesso aos serviços de saúde bucal
	Proporção de consultas agendadas realizadas na UBS
	Proporção de gestantes com atendimento odontológico realizado
	Razão entre atendimentos de demanda espontânea e consultas agendadas
	Proporção de atendimentos odontológicos de urgência na demanda espontânea
	Taxa de atendimentos odontológicos de urgência por população cadastrada
	Média de consultas de retorno em odontologia por tratamento concluído
	Cobertura de primeira consulta odontológica programática
	Razão entre tratamento concluído e primeiras consultas odontológicas programáticas
	Taxa de atendimentos de cirurgiões-dentistas por população cadastrada
Vigilância em saúde bucal	
	Taxa de atendimento odontológico por dor de dente
	Proporção de usuários com dor de dente atendidos na urgência
	Taxa de atendimento odontológico por abscesso dentoalveolar
	Taxa de atendimento odontológico por alteração em tecido mole
	Taxa de atendimento odontológico de usuários com fendas ou fissuras labiopalatais
Diagnóstico, tratamento e reabilitação em saúde bucal	
	Proporção de atendimentos odontológicos de urgência
	Proporção de procedimentos odontológicos clínico-cirúrgicos
	Proporção de procedimentos odontológicos restauradores
	Proporção de exodontias de dentes permanentes nos procedimentos odontológicos
	Média de procedimentos de adaptação por prótese instalada
	Razão entre agendamentos para outros profissionais da APS e atendimentos realizados pela eSB
	Média de encaminhamentos para cirurgia BMF
	Média de encaminhamentos para endodontia
	Média de encaminhamentos para estomatologia
	Média de encaminhamentos para implantodontia
	Média de encaminhamentos para odontopediatria
	Média de encaminhamentos para ortodontia/ortopedia
	Média de encaminhamentos para periodontia
	Média de encaminhamentos para prótese dentária
	Média de encaminhamentos para radiologia
	Apoio diagnóstico em radiologia para procedimentos odontológicos
Promoção e prevenção	
	Proporção de procedimentos preventivos individuais em saúde bucal
	Agendamento de usuários para atividades em grupos pela Equipe de Saúde Bucal
	Atividades de atendimento em grupo
	Atividades de educação em saúde
	Atividades de avaliação/procedimento coletivo
	Ações educativas para crianças na primeiríssima infância (0 a 3 anos)
	Ações educativas para crianças em idade pré-escolar (4 e 5 anos)
	Ações educativas para crianças em idade escolar (6 a 11 anos)
	Ação educativa para controle do tabagismo
	Taxa de participação dos usuários em atividades de educação em saúde
	Taxa de participação dos usuários nos atendimentos em grupo
	Taxa de participação dos usuários nas atividades de avaliação/procedimento coletivo
	Práticas coletivas em saúde bucal
	Temas em saúde bucal nas atividades coletivas
Gestão da saúde bucal	
	Atuação intersetorial/participação popular
	Proporção de reuniões para planejamento e avaliação participativa
	Proporção de atividades de mobilização social
	Grau de participação social em relação às atividades de educação em saúde
Proporção de atividades coletivas direcionadas a profissionais de educação	
	Processo de trabalho da eSB
	Grau de protagonismo das eSB nas reuniões de equipe
	Grau de organização das eSB em relação ao processo de trabalho da equipe
	Grau de organização das eSB em relação às questões administrativas/funcionamento
	Grau de organização das eSB em relação ao diagnóstico e monitoramento do território
	Grau de organização das eSB em relação à discussão de caso e de projeto terapêutico singular
	Grau de organização das eSB em relação à educação permanente

* As fichas de qualificação de cada indicador podem ser consultadas no Dicionário de Indicadores (https://pergamum.bu.ufmg.br/pergamumweb/vinculos/00002d/00002d44.pdf).

UBS: unidade básica de saúde; APS: Atenção Primária à Saúde; eSB: equipe de saúde bucal; BMF: bucomaxilofacial.

### Painel de indicadores

O painel de indicadores, registrado no Instituto Nacional de Propriedade Industrial (BR 51 2023 002193-8), está disponível para implementação no link https://lookerstudio.google.com/reporting/86c09403-f4a0-4625-ad1f-239daa77f6a2/page/p_rpxxv2ub8c30. Destaca-se que há possibilidades de integrar o MonitoraSB a outros sistemas de monitoramento vigentes no Brasil. O painel está em processo de incorporação pela Coordenação Geral de Saúde Bucal do Ministério da Saúde (CGSB) como ferramenta a ser recomendada para o monitoramento dos serviços de saúde bucal no Brasil e será hospedado na Sala de Gestão Estratégica (LocalizaSUS/SEIDIGI). Espera-se também que indicadores da matriz avaliativa componham o painel de saúde bucal integrando-se ao sistema de monitoramento da APS, nos painéis em construção.

O painel possui barras de navegação na página inicial e na barra lateral, possibilitando navegação rápida e interativa de um indicador para outro (Figura 1). A navegação pelo indicador selecionado pode ser realizada obtendo-se uma visão por região ou estado, de acordo com o filtro temporal desejado. Por exemplo, a Figura 2 ilustra os resultados do indicador "Cobertura de primeira consulta odontológica programática" para o ano de 2023, quadrimestre 1.

**Figura 1:** Estrutura de navegação do painel de indicadores para o monitoramento dos serviços de saúde bucal na Atenção Primária à Saúde.

**Figura 2:** Visualização dos resultados do indicador "Cobertura de primeira consulta odontológica programática" com filtros geográficos por região e unidades da federação brasileiras e filtros temporais.

A visualização demonstra os resultados para o Brasil (12 atendimentos odontológicos por mil usuários) e para cada região, com valores variando de 13,5 no Nordeste a 10,4 no Sudeste (por mil usuários). O mapa do Brasil mostra os valores do indicador para cada estado, representando uma escala de cor do menor para o maior valor. Os resultados podem também ser visualizados para cada UF em gráficos de barra, permitindo comparar e analisar as disparidades geográficas nos indicadores. Por exemplo, para esse indicador, os menores resultados foram observados em Roraima e no Acre, e os maiores em Alagoas (Figura 2a). Uma visão detalhada pode ser vista selecionando-se determinada região (Figura 2b). Quando mais de um quadrimestre for selecionado, o painel também possibilita comparações temporais, ou seja, variações do indicador ao longo do tempo, segundo o recorte geográfico selecionado. Por exemplo, a Figura 2b mostra a variação do indicador desde o primeiro quadrimestre de 2020 para cada uma das UFs da Região Sudeste (Figura 2b). Nesse caso, observam-se menores valores do indicador no segundo quadrimestre de 2020, coincidente com a interrupção dos serviços odontológicos em razão da pandemia de COVID-19. A análise temporal pode ser útil para avaliar tendências, efeitos de ações, políticas e programas de saúde e para o planejamento de recursos. Ela é essencial para monitorar os serviços e entender seu funcionamento e resposta durante eventos como a pandemia ou outras situações que demandem resiliência e capacidade de adaptar e reorganizar os serviços.

A análise por município possibilita comparar os indicadores de acordo com características geográficas, demográficas ou socioeconômicas (Figura 3). Por exemplo, a Figura 3 ilustra os resultados do indicador "Cobertura de primeira consulta odontológica programática" para municípios de Minas Gerais com até 5 mil habitantes e Índice de Desenvolvimento Humano Municipal (IDHM) muito baixo. Nesse exemplo, foram selecionados os períodos entre os quadrimestres de 2020Q1 a 2023Q1 para avaliação temporal, evidenciando um padrão semelhante de redução do indicador em 2020Q2 e muitas flutuações, mas, com um padrão de aumento do número de primeiras consultas odontológicas programáticas para cada mil usuários cadastrados em muitos municípios após 2020Q2.

**Figura 3:** Visualização dos resultados da variação temporal do indicador "Cobertura de primeira consulta odontológica programática" no nível municipal, considerando filtros de porte populacional e Índice de Desenvolvimento Humano Municipal.

Observou-se também que o indicador não foi calculado em alguns municípios em 2020Q1 e 2020Q2, fato evidenciado pela interrupção da linha temporal. Nesses municípios e quadrimestres, não houve registro de primeira consulta odontológica programática. O mapa de calor demonstra em cores próximas de verde os municípios com maior cobertura e em vermelho aqueles com os menores valores do indicador. Cabe destacar que no mapa aparece o indicador calculado considerando o período selecionado, no exemplo, para todos os quadrimestres. Além disso, o diâmetro do círculo representa o porte populacional do município. O filtro de IDHM poderia ser usado para análise comparativa dos municípios com outros valores de IDHM (Figura 3a).

Uma análise pode ser realizada selecionando-se somente o município. No exemplo, Alvorada de Minas apresentou valores do indicador menores nos períodos entre 2020Q1 e 2021Q1, com variação positiva desde esse quadrimestre (Figura 3b). O painel também é um gerador de relatórios e base de dados, pois possibilita o *download* dos indicadores calculados em planilhas para os níveis de desagregação Brasil, região, UF e município.

### Calculadora de indicadores

A calculadora de indicadores de saúde bucal é uma ferramenta *online* de acesso aberto (bit.ly/calculadora-indicadores-saude-bucal-ppsus). Acessando a calculadora, o usuário selecionará o indicador que deseja calcular, em seguida, aparecerão na tela caixas para entrada dos dados do numerador e do denominador de cada indicador. Esses dados deverão ser obtidos nos relatórios gerenciais do e-SUS APS para cada eSB. Depois de inserir os dados e clicar em "Calcular Indicadores", os resultados serão exibidos. O usuário terá ainda a opção de fazer *download* dos resultados em formato Excel ou *csv (Figura 4).

**Figura 4:** Visualização das etapas para cálculo de indicadores de saúde bucal usando a calculadora.

### MonitoraSB: implicações para o monitoramento e a avaliação dos serviços de saúde bucal

Este trabalho apresenta uma proposta inovadora de monitoramento e avaliação dos serviços públicos de saúde bucal ao elaborar uma matriz de indicadores e propor duas ferramentas digitais inéditas de acesso livre para operacionalizá-la. A matriz, baseada em dados nacionais de produção em saúde, garante a disponibilidade das informações^36^, orientando decisões políticas e subsidiando o controle social. Os indicadores abordam diversos aspectos do cuidado em saúde bucal desde a cobertura dos serviços na APS, tanto individuais como coletivos, e permite compreender a demanda por serviços especializados, o modelo de atenção predominante e conhecer taxas de atendimentos para agravos específicos^29^.

Ao ampliar o escopo de indicadores, referentes ao provimento e gestão da saúde bucal, a matriz oferece novas possibilidades comparadas às iniciativas existentes até então, que se concentraram em dados relacionados ao acesso e à cobertura de serviços de saúde bucal e que foram reduzidos ao longo dos anos^18,21^.

As ferramentas digitais operacionalizam o cálculo de indicadores e seu uso pelos serviços. Por permitir recortes temporais e espaciais, o painel facilita a comparação dos resultados em múltiplos níveis e favorece o monitoramento contínuo dos serviços de saúde bucal, permitindo a avaliação, a transparência e a reorientação contínua de intervenções.

Como se utiliza dos sistemas de informação do SUS, oferece uma perspectiva baseada na realidade, possibilitando também a qualificação dos registros. Essa ferramenta também fortalece o uso dos sistemas de informação em saúde, alinhando-se à implementação do SUS Digital Brasil^23^. Por incluir dados alimentados por todos os municípios brasileiros e se tratar de uma ferramenta aberta, o painel MonitoraSB democratiza o acesso à informação.

Como utilizam o *Delta Lake* em sua arquitetura para extração e armazenamento dos dados, as ferramentas digitais favorecem um ecossistema inovador para o armazenamento de grande volume e múltiplas formas de processamento das informações^37,38^. Isso favorece a tempestividade das informações acessadas pelos usuários por meio da disponibilização de novos dados pelo SISAB e garante que as informações certas estejam disponíveis no momento e no local certos para uma tomada de decisão baseada em evidências^39^.

Com interface amigável, o painel permite rápida visualização dos resultados e múltiplas comparações temporais e por região, porte populacional e faixas do índice de desenvolvimento humano municipais. Assim, a ferramenta oferece certo grau de customização e recursos visuais automáticos após a seleção do indicador. Considerando a sala de situação em saúde como espaço para análise sistemática das informações^40^, essa ferramenta pode ser entendida como tal ou ainda como apoio às equipes técnicas locais, proporcionando o acompanhamento contínuo dos indicadores para o planejamento e a rápida identificação de ocorrências fora do padrão^41^, auxiliando na vigilância em saúde bucal. Além da customização existente, é possível calcular indicadores individualmente com a calculadora digital, permitindo recortes mais específicos. Essas ferramentas ajudam a superar a fragmentação dos dados em saúde bucal e buscam equidade no acesso à informação e a tecnologias. Esses aspectos são essenciais para melhorar a APS, aumentar sua resiliência, especialmente em um país com grandes desigualdades no uso e no acesso às tecnologias por profissionais e onde a exclusão digital ainda é proeminente^2^.

No entanto, há alguns desafios a serem enfrentados, dentre eles, a necessidade de redimensionamento da matriz de indicadores, algo que o uso das ferramentas em contextos reais deve facilitar. Por outro lado, o escopo ampliado dos indicadores pode contribuir para a mudança dos modelos de monitoramento e avaliação até então existentes no Brasil, que estão frequentemente vinculados ao cumprimento de metas atreladas ao repasse financeiro.

Nessa perspectiva, o MonitoraSB apresenta possibilidades de integração com sistemas de monitoramento existentes, especialmente por meio da CGSB e da SEIDIGI, conforme já mencionado. Além disso, algumas de suas características, como o uso da ciência de dados em seu desenvolvimento e a possibilidade de obtenção das informações por município em formato *csv, também favorecem a interoperabilidade com iniciativas como o PROADESS, por exemplo. Nesse caso, uma das possibilidades é o uso de indicadores de desempenho do sistema de saúde contendo informações geoespaciais para a melhor compreensão dos indicadores de saúde bucal^42^.

Outro desafio é a fonte dos dados, à qual se submete a qualidade dos registros dos sistemas de informação, que podem conter inconsistências, informações conflitantes, dados faltantes ou mesmo ser afetados por sub-registros. Dada ainda sua natureza, o menor nível de desagregação dos indicadores no painel é o municipal. No entanto, a grande maioria dos municípios brasileiros são de pequeno porte, os quais, em torno de 70%, possuem menos de 20 mil habitantes, e ferramentas como essa podem oferecer avanço significativo para sua gestão^43^. A calculadora buscou superar essa limitação.

A adoção de ferramentas digitais nos serviços de saúde da APS é um desafio a ser superado. Uma pesquisa de implementação em contextos municipais está em andamento para avaliação da aceitabilidade, adoção, viabilidade, adequabilidade e sustentabilidade do uso do MonitoraSB pelos profissionais do SUS. Nesta pesquisa, também se analisou seu impacto nos desfechos dos serviços de saúde, observando a variação dos próprios indicadores ao longo do tempo e a qualidade de registro dos dados no e-SUS APS. Espera-se também que o uso dos indicadores em diferentes realidades permita discutir e definir parâmetros de qualidade aceitáveis ou desejáveis para sua mensuração.

O MonitoraSB fornece indicadores para monitorar a saúde bucal dos usuários do SUS, melhorando o planejamento em saúde e contribuindo para o avanço das políticas públicas. Ele fornece informações dinâmicas e em tempo real para gestores, acadêmicos, movimentos sociais e tomadores de decisão. Espera-se que a implementação do MonitoraSB promova o fortalecimento da APS e da PNSB, contribuindo para consolidar um modelo de cuidado em saúde bucal universal, integral e equânime.
